# Self-Assembled Behavior of Ultralightweight Aerogel from a Mixture of CNC/CNF from Oil Palm Empty Fruit Bunches

**DOI:** 10.3390/polym13162649

**Published:** 2021-08-10

**Authors:** Dian Burhani, Athanasia Amanda Septevani, Ruby Setiawan, Luthfia Miftahul Djannah, Muhammad Andrew Putra, Sukma Surya Kusumah, Dewi Sondari

**Affiliations:** 1 Research Center for Biomaterial, Indonesian Institute of Sciences, Cibinong 16911, Indonesia; sukma.surya@biomaterial.lipi.go.id (S.S.K.); dewi.sondari@lipi.go.id (D.S.); 2 Research Center for Chemistry, Indonesian Institute of Sciences, Serpong 15314, Indonesia; athanasia.amanda.septevani@lipi.go.id; 3 Research Center for Biology, Indonesian Institute of Sciences, Cibinong 16911, Indonesia; ruby.setiawan@lipi.go.id; 4 Bioprocess Department, Brawijaya University, Malang 65145, Indonesia; luthfiah8080@gmail.com (L.M.D.); andrewputra11@gmail.com (M.A.P.)

**Keywords:** aerogel, CNC, CNF, freeze-drying, OPEFB

## Abstract

This study aims to explore the use of cellulose nanocrystals (CNC) and cellulose nanofiber (CNF), obtained from unbleached fiber of oil palm empty fruit bunches (EFB), as raw materials in fabricating aerogel, using the facile technique without solvent displacement. The CNC was isolated from sulfuric acid hydrolysis, and the CNF was fibrillated using Ultra Turrax. The CNC and CNF were mixed by ultrasonication in different ratios to produce aerogel using slow freezing (−20 °C), followed by freeze-drying. The obtained aerogel was characterized as ultralightweight and highly porous material, at the density range of 0.0227 to 0.0364 g/cm^3^ and porosity of 98.027 to 98.667%. Interestingly, the ratio of CNC and CNF significantly affected the characteristics of the obtained aerogel. The mixed aerogel exhibited a higher specific surface area than pure CNC or CNF, with the highest value of 202.72 m^2^/g for the ratio of 1:3 (CNC/CNF). In addition, the crystallinity degree of obtained aerogel showed a higher value in the range of 76.49 to 69.02%, with the highest value being obtained for higher CNC content. This study is expected to provide insight into nanocellulose-based aerogel, with a promising potential for various applications.

## 1. Introduction

Aerogel, a highly porous material with ultralightweight density, has been an industrial and scientific interest for the last decade for its unique properties, such as thermal conductivity, flame and moisture resistance, low optical index of refraction, and low dielectric constant [1]. Aerogel mostly consists of air or gas, resulting in a lightweight and high porosity. In addition, it exhibits a mixture of mesopore and macropores structure and a high specific surface area [1]. As a result, aerogel has been widely used in various applications, including oil absorbent [2], water treatment [3], fire-resistant [4], energy storage [5], absorption [6], thermal insulation [7], drug delivery [8], and membrane separation [9].

Silica aerogel was first synthesized by Kistler using sol-gel chemistry, in which the removal of the solvent was conducted through supercritical drying. During the 1970s to 1980s, the generation of metal oxide-based aerogel was developed. In addition, in the early 2000s, the next generation of biomass-based (mostly polysaccharide-based) aerogel began. The biomass-based aerogel showed superior characteristics to the former one. It has low density, high specific surface area, and better mechanical properties than silica-based aerogel [10].

In principle, aerogels are prepared by replacing the solvent in the hydrogel with air without altering the original structure of the material [11,12]. Two methods, namely supercritical drying and freeze-drying, are mostly used for the fabrication of aerogel. Supercritical drying used a low surface tension effect amid the drying step, resulting in aerogel with low density, low shrinkage, and high specific surface area. However, this method is not economical and displays potential safety risks since it uses a high-pressure process that limits its industrial-scale application [13]. Meanwhile, the freeze-drying method is simpler in process and cost-efficient than the supercritical drying. In this method, the liquid was frozen (commonly used freezing temperatures are −20 °C, −80 °C, and −196 °C), and then the liquid is removed by sublimation. The freezing rate before the drying process significantly affects the growth behavior of the ice crystal in designing the microstructure of the aerogel [14].

Cellulose is composed of linear β-(1,4)-d-glucose-polymeric chains. Some of the units of β-(1,4)-d-glucose are assembled to form crystalline cellulose microfibrils [15]. A number of the hydroxyl groups in the cellulose structure monomer forms hydrogen bonds, which acts as a vital role in the physical properties and the packing structure of crystalline cellulose [16]. The packing structure of cellulose significantly contributes to its physical and mechanical properties. For instance, in a crystalline structure, the chain molecules are packed in an orderly manner, resulting in excellent strength and stiffness of cellulose. On the other hand, the disordered regions at the amorphous structure are responsible for the flexibility of cellulose [17].

When subjected to strong acid hydrolysis, macroscopic or microscopic forms of cellulose experiences transverse cleavage at the amorphous regions generating rod-like fiber denoted as cellulose nanocrystals (CNC). Meanwhile, cellulose nanofiber (CNF) can be formed by mechanical fibrillation, including homogenization, microfluidization, and ultrafine grinding. Additional pretreatments, such as enzymatic pretreatment, chemical pretreatment, or mechanical pretreatment, are performed to reduce energy input and improve the quality of the obtained CNF [18].

Both CNC and CNF show specific properties that differentiate their applicability for certain applications [19]. CNC that has a diameter typically around 2–20 nm and lengths from 100 to 600 nm mainly consists of crystalline regions, resulting in high crystallinity [17]. Therefore, CNC is considered superior to CNF in strength, rigid structure, and optical properties [20]. Meanwhile, cellulose nanofiber (CNF) has a diameter of nm and lengths in the micron-scale, showing crystalline and amorphous sections with lower crystallinity than CNC. At low concentration, the long fibrils of CNF can build entangled networks [21], allowing excellent applications, such as functional aerogel and 3D printing [22].

Cellulose aerogel, particularly, has attracted attention due to its ability to be used in various applications. The intrinsic properties of nanocellulose aerogel, such as high surface area, aspect ratio, and abundant hydroxyl groups, for instance, make them suitable for oil recovery [23]. Cellulose aerogel also has better biocompatibility than the traditional aerogel, making it an environmentally friendly absorbent [24,25]. CNC aerogel can match the high porosity and surface area of aerogel based-silica but are much less brittle, making them valuable in applications requiring mechanical flexibility and strength. CNC aerogel also raises interest in the biomedical and pharmaceutical field owing to their accessible pore structure and high specific surface area, promoting drug bioavailability and improved drug-loading capacity [26]. Meanwhile, CNF aerogel is very stable and is easier to prepare than CNC aerogel. The remarkable mechanical strength, high porosity, and flexibility of CNF aerogel allowing them as solid supports for liquid electrolytes in dye-sensitized solar cells and supercapacitor materials. Moreover, more various post-treatments for surface modification have been studied with this material, broadening the potential uses of CNF aerogel [21].

Nanocellulose-based aerogel was first fabricated by Pääkkö et al. from CNF using freeze-drying. The obtained aerogel had high porosity of 98.7% and displayed excellent mechanical properties [13]. Heath and Thielemans (2010) prepared cellulose nanowhiskers’ aerogels from cotton wool using supercritical CO_2_ drying and obtained aerogel with densities down to 78 mg/cm^3^ with high specific surface areas up to 605 m^2^/g [27]. Shamskar et al. (2016) fabricated CNC aerogel via freeze-drying from raw cotton and cotton stalk and obtained aerogels with specific surface area of 91.47 and 93.89 m^2^/g, respectively [28]. Currently, most studies are focusing on the preparation of aerogel by either CNC [28,29,30,31,32] or CNF [33,34,35,36,37,38,39,40] only. A report focusing on the development of aerogel by mixing these two types nanocellulose is scarce. Zhang et al. (2018) combined CNC and CNF from eucalyptus wood pulp. The mix aerogel was fabricated by freeze-drying in combination with solvent replacement using tert-butyl ethanol. The aerogel exhibited high specific surface area and compressive strength compared to pure CNC or CNF aerogel [41].

Besides the isolation process, raw material also significantly affects the properties of the obtained nanocellulose and its aerogels. Currently, nanocellulose-based aerogels have been fabricated from various sources of biomass, including rice straw [42], jute fibers [43], *Eucalyptus* sp. and *Pinus eliottii* [37], poplar wood and cotton [44], softwood [38], pine needles [45], bamboo [12], kapok [46], and needle wood [47]. Indonesia, one of the largest palm oil producers globally, produced more than 45,000 tons of oil palm in 2019 [48]. The palm oil production generates solid residue referred to as empty fruit bunch (EFB), accounting for 20% of fresh fruit weight [49]. One ton of crude palm oil (CPO) generates around 1.2 tons of EFB [50]. When not handled properly, this enormous amount of waste could potentially pose a harmful threat to the environment. Oil palm empty fruit bunch contains a considerable amount of cellulose, around 40–45% [51]. Therefore, when managed correctly, the sustainability of the product based-EFB is ensured.

The isolation of nanocellulose from oil palm empty fruit bunches has been conducted by various methods [50,52,53]. However, no study of the preparation and characterization of aerogel-based nanocellulose from oil palm empty fruit bunches has been reported to the author’s knowledge. This study aims to utilize the CNC and CNF obtained from the unbleached fiber of oil palm empty fruit bunches in fabricating aerogel, using the freeze-drying method without the addition of chemical solvents. The use of unbleached cellulose could be beneficial to the fabrication of aerogels since there is no bleaching process is required leading to less chemical waste. The CNC was isolated using sulfuric acid hydrolysis. Meanwhile, the CNF was prepared from mechanical treatment using Ultra Turrax. The morphology, self-assembly, and crystallinity degree of the obtained aerogel were investigated.

## 2. Materials and Methods

### 2.1. Materials

The EFB for the raw material in this study was obtained from PT. Perkebunan Nusantara II (PTPN II, North Sumatra, Indonesia). The EFB was chopped to 30 mesh and oven-dried until the moisture content was less than 10%. The dried EFB was delignified with 10% NaOH (technical grade) (solid to liquid ratio of 1:5) at 150 °C for 30 min in CHEMEX (Chemical Explosion) reactor (custom made by KOICA, Seongnam, South Korea). The pressure was controlled at 4 bar in the beginning and maintained at 7–8 bar. The delignified EFB was neutralized to pH 7 and oven-dried until the moisture content reached ~10% [50] and further proceeded (without bleaching process) to acid hydrolysis and mechanical treatment to produce cellulose nanocrystals (CNC) and cellulose nanofiber (CNF), respectively. The acid hydrolysis of CNC [54] and mechanical treatment of CNF was conducted from previous studies. The acid hydrolysis used H_2_SO_4_ (Merck, Darmstadt, Germany) 36% with a solid/liquid ratio of 1:80. The obtained CNC was then neutralized by simultaneous centrifugation and dialysis. Meanwhile, CNF was produced by mixing the unbleached cellulose with aquadest with a solid to liquid ratio of 1:185 using Ultra Turrax (Heidolph DIAX 600, Heidolph, Schwabach, Germany) at 20,000 rpm. No additional chemicals were used in the fabrication of aerogel.

### 2.2. Methods

#### 2.2.1. Fabrication of Aerogel-Based CNC/CNF Mixture

A 2%wt aqueous suspension of CNC/CNF mixture was prepared with a mass ratio of 1:1, 1:2, 1:3, 2:1, and 3:1 using Ultrasonication (Sonic Ruptor 400, Omni Inc., Kennesaw, GA, USA). The mixtures were frozen at −20 ± 1 °C for 18 h. The frozen samples were freeze-dried (FreeZone, Labconco, Kansas city, MO, USA) at a temperature of −50 ± 2 °C under a vacuum of 0.1–0.05 mbar for 48 h to produce aerogel, named A-C/F_xx_, where x denotes a ratio of nanocellulose. For example, A-C/F_13_ was aerogel from a mixture of CNC and CNF with a ratio of 1:3.

#### 2.2.2. Characterization

Density and porosity of aerogel were calculated as follows:

(1) $$\mathrm{porosity}\;\left(\%\right)=\left(\frac{\rho_{c}-\rho_{a}}{\rho_{c}}\right)\times 100$$

where $\rho_{a}$ and $\rho_{c}$ are the bulk density of cellulose taken as 1.69 g cm^−3^ and aerogel density, respectively. The calculation of the aerogel density ($\rho_{a}$ = mass/volume), and the mass and dimensions (diameter and height) of each cylindrical CNF aerogel were measured using a digital caliper [55]. The FTIR spectra of the obtained aerogel for 1:1, 1:2, and 3:1 mixing ratios of CNC/CNF were recorded on a Fourier-transform infrared instrument (PerkinElmer Spectrum Two, Waltham, MA, USA) in 400–4000 cm^−1^. The FTIR spectra of all samples were collected using the attenuated total reflection technique (ATR). Self-assembly structure was observed using a Surface Electron Microscope (SEM) Hitachi TM3030 (Hitachi, Tokyo, Japan) with an acceleration voltage of 15 kV. Specific surface areas were determined by the Brunauer–Emmett–Teller (BET) method using N_2_ adsorption/desorption measurements (Quantachrome Nova 4200e) (Quantachrome, Florida, USA) at −196 °C. Each sample was degassed at 70 °C overnight and then at 115 °C for 4 h. Thermal properties of the aerogel were performed on a TGA-4000 PerkinElmer (PerkinElmer, Waltham, MA, USA). Each sample (10 mg) was heated at 10 °C/min from 25 °C to 500 °C under purging N_2_ (40 mL/min). Crystallinity degree was characterized by using X-ray Diffraction (XRD) Shimadzu X (Shimadzu, Kyoto, Japan) 7000 at 40 kV and 30 mA within 2°–40°. Crystallinity (%) was calculated using Segal’s method as follows:

(2) $$\%\;\mathrm{CrI}=\frac{\;I\left(200\right)-I\left(amorphous\right)}{I\left(200\right)}\times 100\;\%$$

where *I*(200) is the (height) intensity of crystallinity peak at the maximum 2θ between 22°–23°, and *I*(*amorphous*) is the minimum peak (height) intensity at 2θ between 18°–19° (of the amorphous region). The morphology of CNC and CNF was analyzed using JEOL JEM 1010 (JEOL, Tokyo, Japan) at 80 kV.

## 3. Results and Discussion

**Figure 1:**
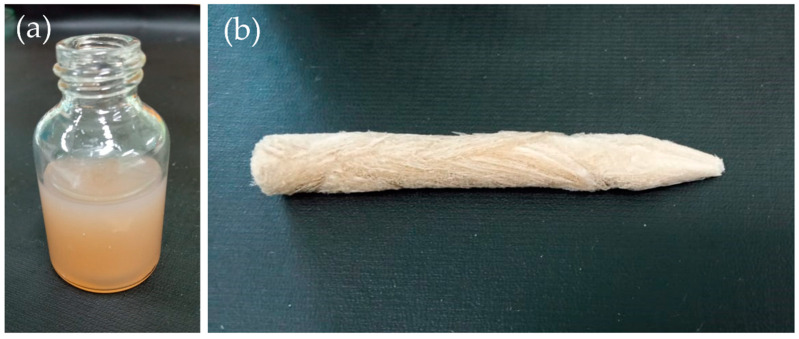
Photograph of aerogel CNC/CNF mixture (**a**) suspension; (**b**) aerogel.

Ultralightweight aerogel with high porosity had been prepared from the mixture of CNC/CNF with different ratios via freeze-drying. The aerogel was cylindrical, shaped by a Corning tube, and it could be easily sliced using a sharp razor. However, it was also fragile and easily deformed with a simple pinch gesture. The unbleached nanocellulose (CNC and CNF) affected the color of the aerogel to light brown (see Figure 1) since they still contained lignin and hemicellulose with a concentration of 12.1 ± 0.5% and 14.9 ± 0.2%, respectively [51]. From Figure 1, we can see that the aerogels exhibited rougher surfaces and streaks, indicating the area where ice crystals formed, which was commonly observed from aerogel fabricated by the freeze-drying process.

**Figure 2:**
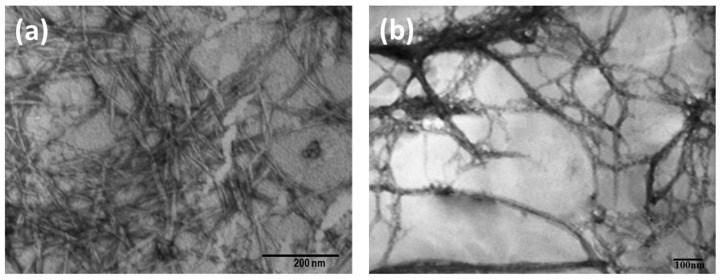
TEM Image of (**a**) CNC from acid hydrolysis (with permission from Carbohydrate Polymers) and (**b**) CNF from Ultra Turrax.

The morphology of the original CNC and CNF was analyzed using TEM. As previously reported from our previous work [54], the diameter and length of the original CNC were 9.6 ± 2.5 nm and 123.3 ± 15.3 nm, respectively (Figure 2a). Meanwhile, the long-entangled CNF was observed from Figure 2b, with the diameter ranging from 10 to 25 nm with length in micrometer. Meanwhile, entanglement of the microfibers of the nanocellulose is a significant factor in building a 3D network of aerogel. Therefore, we used CNF aerogel only and CNC aerogel only as a comparison. However, the short and rigid-structured of CNC [56] turned to powder and failed to form a 3D network structure due to limited entanglement and weak hydrogen bonding [13,57]; therefore, no CNC aerogel was reported in this study.

Table 1 represents densities and porosities from aerogel of CNC/CNF mixtures with different ratios. The density of the obtained aerogels was measured in the range of 0.0227 to 0.0364 g/cm^3^, suggesting its ultra-low density. Meanwhile, the average porosities of aerogel are also relatively high, ranging from 98.027 to 98.667%. It is noted that, when the mass ratio of CNC was fixed and the CNF was varied, the density was increased. On the other hand, when the mass ratio of CNF was set and the CNC was varied, no significant change was observed in the density value. A similar phenomenon was also noticed in porosity. The feasible explanation is that, when there are more CNF than CNC in the suspension, CNF characters play a more important role and vice versa. CNF with long fibers might tend to aggregate more than CNC and formed more entanglement. Therefore, the increase of CNF concentration reducing the space between the fibers resulted in a continuous decrease of porosity. This was supported by the SEM image in Figure 5, in which smaller pore size and lamellar structure were more prominent in Figure 5a–c.

Meanwhile, lower density was observed when the CNC mass portion is bigger than CNF. This might be attributed to the character of CNC, which has good dispersion, leaving more voids to fill the air, allowing low density and high porosity, which was supported by SEM image in Figure 5, in which larger pore size was more prominent in Figure 5d,e.

### 3.1. Chemical Structure

**Figure 3:**
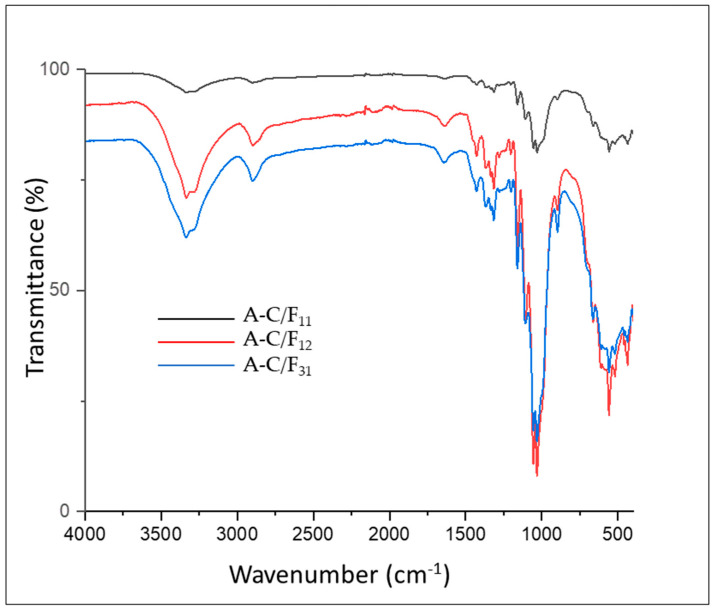
FTIR spectra of aerogel-based CNC/CNF mixture A-C/F_11_, A-C/F_12_, and A-C/F_31._

The changes in chemical structure from the blending process of CNC and CNF in three different ratios were illustrated in Figure 3. All of the FTIR spectra showed similar peaks, indicating no significant changes in their chemical composition with the blending process. However, stronger transmittances were observed when the CNC and CNF mass portions were equal and weaker when the CNC mass portion was bigger.

As shown in Figure 3, the three samples displayed two primary regions of absorbance; the first one at low wavelengths (500–1750 cm^−1^) and the second one at higher wavelengths (2800–3700 cm^−1^) [58]. Overall, all samples exhibit almost similar peaks, indicating no change in the chemical structure of the aerogel. Visible differences were observed from the intensity of several significant peaks. The increase of the CNC mass portion resulted in an increase of the band at 3334 cm^−1^ and 2899 cm^−1^. The -OH stretching and bending vibrations were observed to be stronger and narrower for A-C/F_31_ than the others. Similarly, the band at 2899 cm^−1^ which was attributed to the aliphatic saturated C–H stretching vibration in cellulose, hemicellulose, and lignin, exhibited peak decrease for A-C/F_11_. The absence of shoulder peak at 1726 cm^−1^ in all samples implied no acetyl and uronic ester groups of the hemicelluloses or the ester linkage of lignin [59]. This was in accordance with the lignocellulosic content measurement that showed a decrease of hemicellulose and lignin content after delignification.

Meanwhile, the peak at around 1643 cm^−1^ in all samples indicates water absorption by cellulose [60]. The peaks at 1427–1428 cm^−1^ were stronger and narrower for A-C/F_21_ and A-C/F_31_ than A-C/F_11_, which was attributed to the crystalline band of cellulose, indicating that the A-C/F_21_ and A-C/F_31_ have higher crystallinity degree than A-C/F_11_. The peaks at 1335–1369 cm^−1^ present in all samples are assigned to the bending vibration of the C-H and C-O groups of the aromatic ring in polysaccharides [61]. The appearance of peaks at 1203–1054 region cm^−1^ reveal the C–O stretch band and deformation bands in cellulose, lignin, and residual hemicelluloses [62]. The peaks at 1160 cm^−1^ observed in all samples, attributed to the presence of a sulfated group (SO_2_) from the CNC portion because of the sulfonation of cellulose during the hydrolysis process using sulphuric acid [63]. The peaks were stronger and narrower for A-C/F_31_ since the aerogels contained more CNC mass portions. Finally, the peak at 896 cm^−1^ observed in all samples reveals the typical cellulose structure (due to β-glycosidic linkages of glucose ring of cellulose) and C-H rocking vibrations of cellulose [62].

### 3.2. Morphology

**Figure 4:**
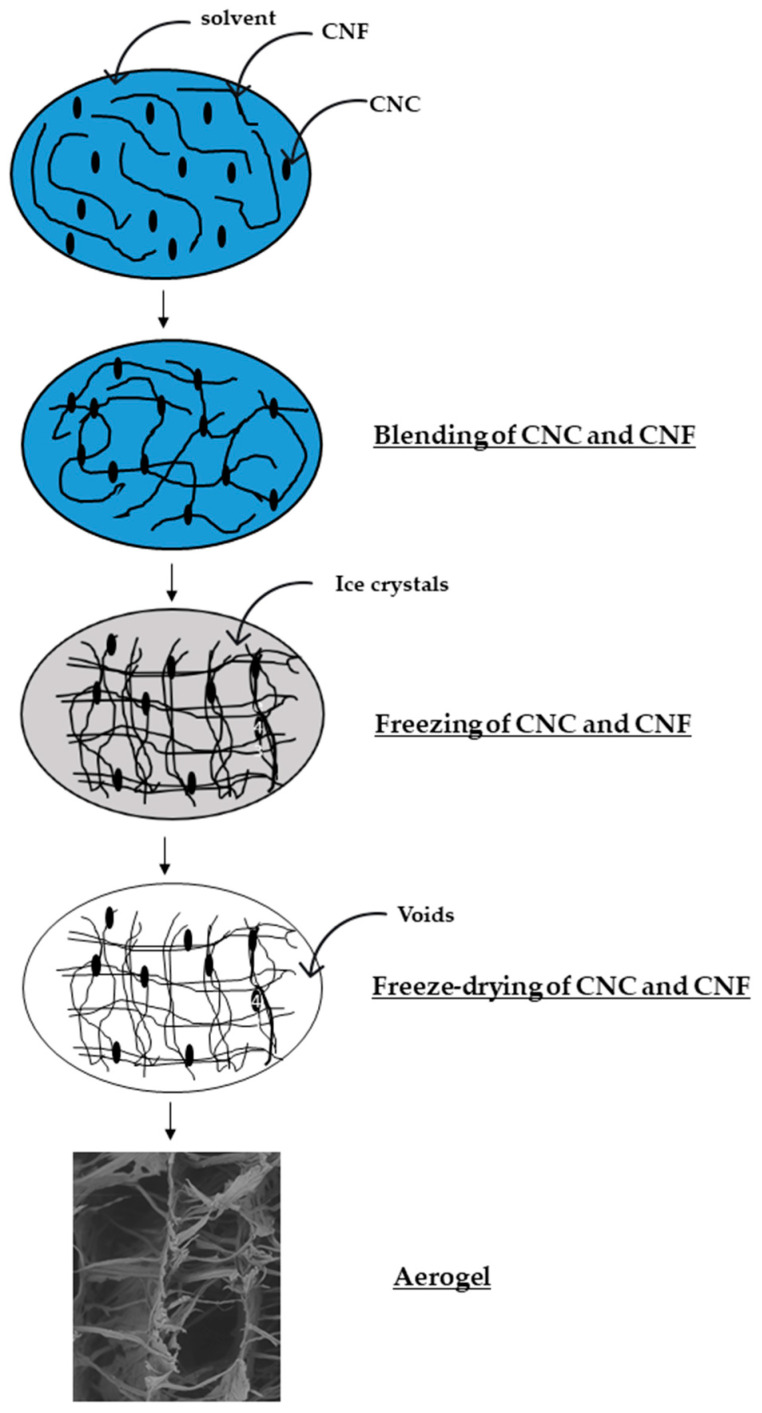
Hypothesis schematic of the fabrication of CNC/CNF aerogel.

CNC and CNF formed the 3D structure of aerogel in different manners. It is quite challenging for CNC, with its rigid structure and weak hydrogen bonding, to form aerogel. Therefore, chemical crosslinking is often used to improve its structural integrity and stability [64]. Meanwhile, CNF with long fibrils can more easily establish the 3D network by the entanglement of the fibrils’ strong hydrogen bonding [65]. Figure 4 illustrates what might happen during the fabrication of aerogel. The use of Ultra Sonication improves the dispersion of CNC and CNF. The blending of CNC and CNF was occurred by entanglement and self-assembly without the use of a cross-linker. The main principle of the fabrication of aerogel was freezing and sublimation. The growing ice crystals from the freezing stage will turn to void, leaving a stable 3D structure.

**Figure 5:**
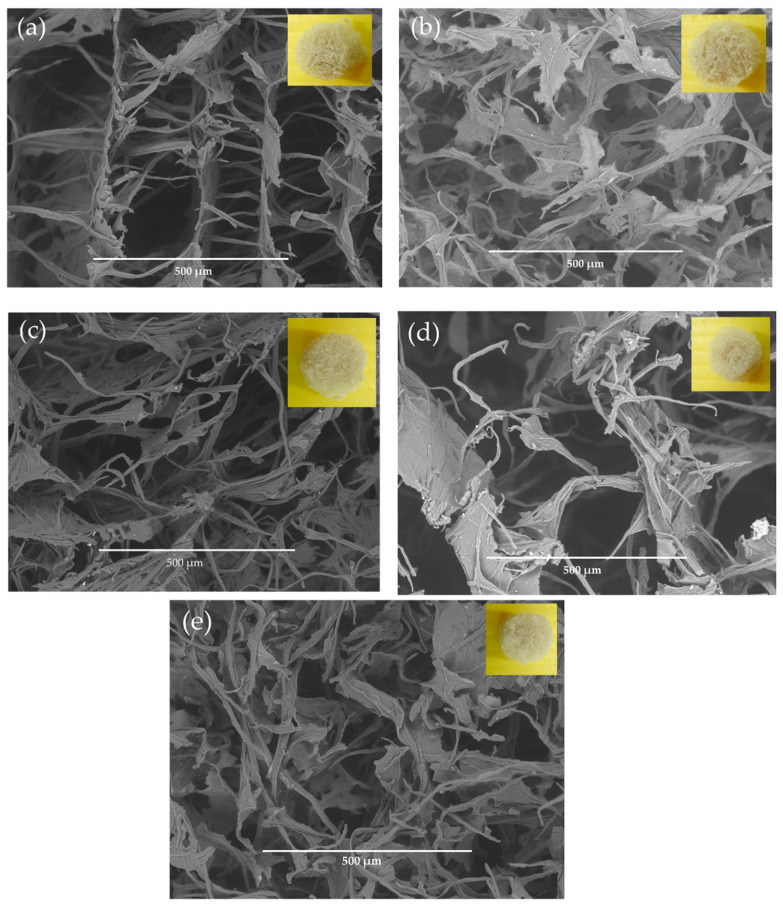
SEM analysis of aerogel-based CNC/CNF mixture (**a**) A-C/F_11_; (**b**) A-C/F_12_; (**c**) A-C/F_13_; (**d**) A-C/F_21_; (**e**) A-C/F_31._

Figure 5 illustrates the SEM image of aerogels, which shows the entangled network of nanofiber. The fiber bundle size was measured using ImageJ software (v.1.49, National Health and LOCI, University of Wisconsin, Madison, WI, USA), and the result showed that the diameter of the fiber bundle ranged from 1 to 2 µm. The morphology of the aerogel was investigated via SEM. The prepared aerogels showed various thin sheets, sheet-like skeleton structures, and compact film-like structures, which was common to observe when the aerogel formed during a relatively slow freezing process (−20 °C). This phenomenon could be associated with strong hydrogen bonding, which brings the nanofibrils together into bigger-sized bundles or film-like structures [66]. Figure 5a–c, of which the ratio of CNF was higher than CNC, show a thinner sheet-like skeleton. Figure 5d,e, of which the ratio of CNC was higher than CNF, exhibit a compact film-like structure and bundle structure. This phenomenon was probably attributed to the freezing process in which the CNF suspension was aggregated at the edge of the growing ice crystals. The aggregated CNF might be bounded through hydrogen bonding and or entanglement. Finally, sheets were formed and maintained after the removal of the solvents [23].

The concentration of the suspension can also influence the structure of aerogel. This study used a relatively high concentration of nanocellulose, which was 2 wt% (total concentration of CNC/CNF suspension). The concentration was quite high compared to other studies. Corresponding to this, Chen et al. (2014) observed aerogels obtained with various concentrations. The study found that, at high concentrations (>0.5 wt%), there is probably not enough space for nanocellulose to disperse. As a result, after it freeze-dried, the nanocellulose firmly cross-linked to each other and ultimately formed 2D-sheet structures [44]. This justification was also backed up by Han et al. (2013), who found that the self-organizing characteristic of nanocellulose in suspension significantly depends on the arrangement of hydrogen bonds. Furthermore, less space between the fibers during the freezing process, at nanocellulose concentration > 2 wt%, allows the formation of intra- and intermolecular hydrogen bonds and Van der Waals forces arranging them in a longitudinal direction, generating a lamellar structure [67].

Various sizes and shapes of pores were observed between the sheets of the aerogel. Contrary to the mesopores (2–50 nm) of the aerogel formed by cellulose nanofiber or cellulose nanocrystals [68], highly porous structures of the obtained aerogel with macropores (>100 µm) [69] can be observed from the SEM image in Figure 5. Porous diameters of 300–400 µm were observed from all CNC/CNF mixtures aerogels. The macroporous structure might be formed due to the slow growth of ice crystals at the freezing stage (−20 °C) used in this study, resulting in many large pores after the freeze-drying process [65]. The pore size observed in the aerogels was not uniform, which might be caused by the irregular growth of ice crystals during the freeze-drying process [70]. Apparent and uniform porous was clearly observed in Figure 5a for CNC/CNF mixtures aerogels with a ratio of 1:1 (A-C/F_11_).

The macroporous structure of the obtained aerogel in this study is beneficial, especially in oil absorption or recovery application. The various pore diameter in the macroporous structure formed an open and large porous structure enabling the oil to enter into the aerogel easily and improving the oil absorption [25,71,72].

### 3.3. Specific Surface Area

The specific surface area of the obtained aerogels was calculated from the adsorption isotherms was found to vary between 117.86 m^2^/g to 464.39 m^2^/g (Table 2). The specific surface area of the obtained aerogel is much higher than the mixed aerogel CNC/CNF fabricated by Zhang et al. (2018) from eucalyptus wood pulp using freeze-drying and solvent replacement, which was 143 m^2^/g [41]. The value of the specific surface area was one of the highest reported in the literature, specifically for freeze-drying without any additional solvent or treatment. As shown in Table 2, when the mass portion of CNC was fixed, and CNF was varied, the specific surface area of CNC/CNF aerogel increased. The reason might be caused by the increased portion of long CNF, which improves the entanglement, allowing a more compact 3D network structure. When the mass portion of CNF was set and the CNC was varied, the specific surface area was noted to be much higher than other aerogels but decreased with the increase of the CNC mass portion. It is known that density, porosity, and specific surface area are strongly correlated. However, the prominent high value of the specific surface value of A-C/F_21_ was not in line with the value of the density and porosity value of all the obtained aerogels, which did not show significant differences, and also the fact that the aerogels have macroporous structure. Therefore, we assumed that there was a possibility of the influence of the aerogel’s roughness, leading to increased adsorption area or the feasible penetration of the nitrogen molecules into the aerogel during the analysis that disrupted results as occurred in Buesch et al. (2016) [30]. It was noted that the specific surface area of A-C/F_31_, which has more CNC mass portion, was lower than A-C/F_13,_ which has CNF mass portion. This result was in accordance with Zhang et al. (2018), who also reported a higher specific surface area at a higher CNF mass portion [41].

Specific surface area is a significant characteristic of aerogel, which indicated the effectiveness of the drying process [73]. The high specific surface area of the obtained aerogel expresses a small diameter of nanofibrils and plenty of micropores, which supported the morphology of the aerogels [73,74]. Moreover, aerogels with high specific surface area have a lot of potential for functional carrier application, including electrical devices, catalysis, fuel storage, and drug release [73].

### 3.4. Crystallinity Degree

**Figure 6:**
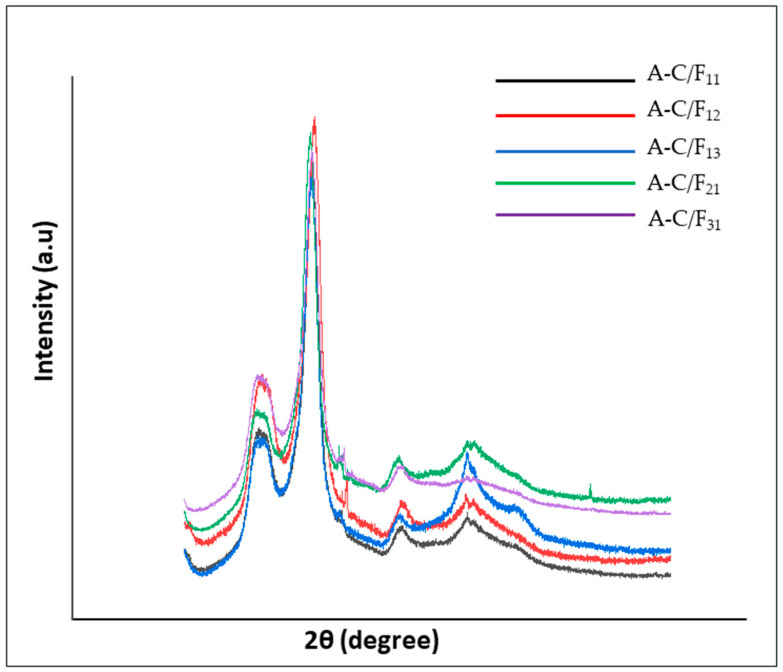
XRD curves of the obtained aerogel.

Crystallinity index (CrI) is defined as the ratio of the crystalline to the amorphous regions of cellulose [63]. The crystallinity degree significantly affects the mechanical and thermal properties of the cellulose [45]. The XRD patterns of the aerogels are shown in Figure 6. The diffraction peaks of all of the obtained aerogel show 2θ = 15°, 22.3°, and 34°, concerning the (1 1 0), (2 0 0) planes and (0 0 4), which are characteristically attributed to the cellulose Iβ structure [75].

Crystallinity degrees of obtained aerogel were 73.06%, 69.02%, 71.78%, 76.49, and 73.15% for A-C/F_11_, A-C/F_12_, A-C/F_13_, A-C/F_21_, and A-C/F_31_, respectively. A similar crystallinity index was reported for cellulose nanocrystal and cellulose nanofiber from oil palm empty fruit bunch of 73% by Lani et al. (2014) [53] and 69% by Jonoobi et al. (2011), respectively [61]. The major crystalline peaks at 22.6° with high intensity were observed for A-C/F_21_ and A-C/F_31_, confirming crystalline cellulose [59]. The results showed that aerogels with a higher ratio of CNC had a higher degree of crystallinity than others. This is attributed to the fact that CNC itself has a higher crystallinity degree than CNF. CNC used in this study was isolated by sulfuric acid hydrolysis. It is known that, during hydrolysis, the amorphous regions of cellulose were attacked by sulfuric acid, leaving behind the crystalline regions, which have greater resistance. The hydrolysis eliminates the microfibrils resulting in cellulose nanocrystals as the final product [50]. Furthermore, the growth and transition of monocrystals might happen simultaneously, adding an increase in the crystallinity of cellulose, and sharpening the diffraction peaks of the XRD graph [63]. Therefore, in the blending process between CNC and CNF, the crystalline allocation to the total crystalline and amorphous ratio decreases, resulting in a lower degree of crystallinity and vice versa.

## 4. Conclusions

CNC/CNF mixture aerogel was successfully fabricated from unbleached cellulose of an oil palm empty fruit bunch by a freeze-drying method without additional treatment or solvent. The macroporous structure with lamellar structure was prominently observed in the obtained aerogels as a result of slow freezing temperature (−20 °C) before the freeze-drying. The obtained aerogel has an ultralight density (0.0225 to 0.364 g/cm^3^), high porosity (97.84 to 98.67%), high crystallinity (69.02 to 76.49%), and high specific surface area (117.86 to 202.72 m^2^/g), indicating that it can store other material, making it have potential as an absorbent as well as other applications, including electrical devices, catalysis, fuel storage, and drug release. From these results, it was found that the ratio of CNC and CNF significantly affected the characteristics of the obtained aerogel. Therefore, tunable aerogel can also be controlled by varying the CNC and CNF content of the aerogel.

## Figures and Tables

**Table 1:** Density and porosity of aerogel-CNC/CNF mixture in different ratios.

Aerogel	CNC/CNF Ratio	Density (g/cm^3^)	Porosity (%)
A-C/F_11_	1:1	0.0277 ± 0.004	98.027 ± 0.0024
A-C/F_12_	1:2	0.0275 ± 0.002	98.364 ± 0.0002
A-C/F_13_	1:3	0.0364 ± 0.0007	97.843 ± 0.0004
A-C/F_21_	2:1	0.0227 ± 0.0009	98.659 ± 0.0006
A-C/F_31_	3:1	0.0225 ± 0.0009	98.667 ± 0.0005

**Table 2:** Single points’ surface area data.

Aerogel	CNC/CNF Ratio	Relative Pressure [P/Po]	Volume @ STP [cc/g]	1/[W ((P/Po) − 1)]	Specific Surface Area (m^2^/g)
A-C/F_11_	1:1	0.2951	41.2105	8.13	126.42
A-C/F_12_	1:2	0.3017	48.7459	7.09	148.15
A-C/F_13_	1:3	0.2972	41.8069	8.094	202.72
A-C/F_21_	2:1	0.3022	75.5596	4.587	464.39
A-C/F_31_	3:1	0.2976	38.5557	29.54	117.86
